# Identification of MBW Complex Components Implicated in the Biosynthesis of Flavonoids in Woodland Strawberry

**DOI:** 10.3389/fpls.2021.774943

**Published:** 2021-11-08

**Authors:** Pengbo Xu, Liang Wu, Minghao Cao, Chao Ma, Kun Xiao, Yanbang Li, Hongli Lian

**Affiliations:** ^1^School of Agriculture and Biology, Shanghai Jiao Tong University, Shanghai, China; ^2^Department of Laboratory Medicine and Pathology, Mayo Clinic, Rochester, MN, United States

**Keywords:** woodland strawberry, MBW complex, flavonoid biosynthesis, MYB, bHLH

## Abstract

Flavonoids belong to the family of polyphenolic secondary metabolites and contribute to fruit quality traits. It has been shown that MBW complexes (MYB-bHLH-WD40) regulate the flavonoids biosynthesis in different plants, but only a limited number of MBW complexes have been identified in strawberry species in general. In this study, we identified 112 R2R3-MYB proteins in woodland strawberry; 12 of them were found to have potential functions in regulating flavonoids biosynthesis by phylogenetic analysis. qRT-PCR assays showed that *FvMYB3*, *FvMYB9*, *FvMYB11*, *FvMYB22*, *FvMYB64*, and *FvMYB105* mostly expressed at green stage of fruit development, aligned with proanthocyanidins accumulation; *FvMYB10* and *FvMYB41* showed higher expression levels at turning and ripe stages, aligned with anthocyanins accumulation. These results suggest that different MYBs might be involved in flavonoids biosynthesis at specific stages. Furthermore, FvMYB proteins were demonstrated to interact with FvbHLH proteins and induce expression from the promoters of *CHS2* and *DFR2* genes, which encode key enzymes in flavonoids biosynthesis. The co-expression of FvMYB and FvbHLH proteins in strawberry fruits also promoted the accumulation of proanthocyanidins. These findings confirmed and provided insights into the biofunction of MBW components in the regulation of flavonoid biosynthesis in woodland strawberry.

## Introduction

Strawberry is favored by consumers mainly because of its unique aroma, sweet taste, bright color and nutritional value. These quality traits are largely determined by the secondary metabolites in the fruit. Flavonoids, which are natural polyphenol compounds, consist of six major subgroups, including chalcones, flavones, flavonols, flavandiols, anthocyanins, and proanthocyanidins (Li, 2014). Flavonoids not only give plants their attractive colors for pollen and seed dispersal, but also protect plants against ultraviolet radiation (Winkel-Shirley, 2001; Emiliani et al., 2013). In addition, flavonoids may act as antioxidants or signaling molecules to exert potential beneficial effects on human health (He and Giusti, 2010), as well as being antimicrobial agents in plant-microbe interactions and plant defense response (Nijveldt et al., 2001; Battino et al., 2009).

Flavonoids are synthesized from malonyl-CoA produced by the fatty acid metabolic pathway and 4-coumaroyl-CoA produced by the phenylpropanoid metabolic pathway with the catalysis by a variety of enzymes. In general, three molecules of malonyl-CoA and one molecule of 4-coumaroyl-CoA were converted into one anthocyanin under a successive catalytic action of many enzymes, including chalcone synthase (CHS), chalcone isomerase (CHI), flavonoid 3-hydroxylase (F3H), flavonol synthase (FLS), flavonoid 3′-hydroxylase (F3′H), dihydroflavonol-4-reductase (DFR), anthocyanidin synthase (ANS), leucoanthocyanidin reductase (LAR), anthocyanidin reductase (ANR), and 3-glycosyltransferase (3-GT). In the synthesis pathway of anthocyanins, the intermediate metabolites dihydrokaempferol and dihydroquercetin, products of F3H, can be oxidized by FLS to produce flavonols. Similarly, the products of DFR and ANS, leucocyanidin, and cyanidin, can also be converted into proanthocyanidins precursors by LAR and ANR, respectively.

Flavonoids biosynthesis has been well studied in many plants and is largely regulated at the transcriptional level by MYB-bHLH-WD40 (MBW) complex. The MYB protein family is one of the largest families of transcription factors in plants. According to the number of R motifs contained in a MYB gene, it could be divided into four subfamilies, namely 1R-MYB with one R domain, 2R-MYB (R2R3-MYB) with two R domains, and 3R-MYB or 4R-MYB with three or four R domains. Among the four subfamilies, R2R3-MYB is the most abundant, which is widely involved in regulating plant growth and development, responses to biotic and abiotic stresses, as well as environmental factors (Dubos et al., 2010). MBW comprises three classes of regulatory proteins, including R2R3-MYBs, bHLHs and TRANSPARENT TESTA GLABROUS1 (TTG1; also termed WD40) (Li, 2014). In *Arabidopsis*, it has been reported that the three regulatory proteins, namely AtTT2/AtMYB123 (Nesi et al., 2001; Dubos et al., 2010), AtTT8/AtbHLH042 (Nesi et al., 2000), and AtTTG1 (WD40-repeat protein) (Walker et al., 1999; Gonzalez et al., 2009), act together as a MBW complex to promote the production of proanthocyanidins in seed coat, by activating the expression of target gene *BAN*/*ANR* (Baudry et al., 2004). The genes involved in the synthesis and accumulation of anthocyanins are also regulated by MBW complexes, comprise at least one R2R3-MYB protein from AtMYB75, AtMYB90, AtMYB113 and AtMYB114, one bHLH protein from TT8, GLABROUS3 (GL3) and ENHANCER OF GLABRA3 (EGL3), and one WD40 protein, AtTTG1 (Dubos et al., 2010; Xu et al., 2015).

For fruit crops, such as grapevine, apple, peach and strawberry, the identification of key MYB proteins have significantly contributed to the understanding of the regulation of fruit flavonoids synthesis (Azuma et al., 2008; Terrier et al., 2009; Zhang et al., 2014; Zoratti et al., 2014; Tuan et al., 2015). Schaart et al. (2013) identified FaMYB9 and FaMYB11 as regulators of proanthocyanidins in octoploid strawberry by a strategy combining yeast-two-hybrid screening and agglomerative hierarchical clustering of transcriptomic and metabolomic data. FvMYB10 has been well studied for its function in the accumulation of anthocyanin in woodland strawberry (Lin-Wang et al., 2014; Castillejo et al., 2020). In the MBW complex, MYB protein is the decisive component for the regulation of flavonoids synthesis. The R2R3 motif at the N-terminal of MYB is responsible for the specific binding to the promoter of target genes, while the C-terminal is responsible for the activation or inhibition of the expression of target genes (Mehrtens et al., 2005; Prouse and Campbell, 2012; Franco-Zorrilla et al., 2014; Kelemen et al., 2015). bHLH protein functions by interacting with MYB (Grotewold et al., 1994). WD40 protein seems to have a more general role in the regulatory complex (Hichri et al., 2010). bHLH proteins in MBW complexes also play important roles in anthocyanin accumulation. There are 133 bHLHs in *Arabidopsis*, three of which have been proven to be related to anthocyanin formation. AtTT8 is a key regulator of anthocyanin and proanthocyanidins biosynthesis. AtEGL3 and AtGL3 mainly act in vegetative tissues (Ramsay and Glover, 2005; Gonzalez et al., 2008). In petunia, the bHLH protein AN1 and JAF13 can interact with AN2 (a R2R3-MYB protein) and WD40 protein AN11, thereby activating anthocyanin biosynthesis genes (Quattrocchio et al., 1993; deVetten et al., 1997; Spelt et al., 2000). In grape, apple and pear, several bHLH proteins, such as VvMYC1, MdbHLH3, MdbHLH33, PpbHLH3, and PpbHLH33, have been found to promote anthocyanin accumulation, and these proteins can form MBW complexes with MYB proteins (Espley et al., 2007; Hichri et al., 2010; Lin-Wang et al., 2014). Similarly, FvbHLH33 can help FvMYB10 to activate the expression of *FvDFR* and *FvUFGT*, key enzyme genes of anthocyanin synthesis. However, there is still limited information available on the transcriptional regulation of the flavonoids biosynthesis by the MBW complexes, especially in the fruits of woodland strawberry.

To identify additional regulatory proteins involved in the flavonoids biosynthesis pathways in woodland strawberry, we first identified the *R2R3-MYB* gene family using latest genome database version 4.0.2a. Novel *MYB* and *bHLH* genes that are possibly related to flavonoids biosynthesis were identified through homology alignment and comprehensive phylogenetic analysis. The corresponding MYB and bHLH proteins were confirmed to form functional complexes using molecular and biochemical experiments. The results from our study contribute to the understanding of flavonoids biosynthesis in the fruits of woodland strawberry at molecular levels.

## Materials and Methods

### Plant Materials and Growth Conditions

*Fragaria vesca* seeds of RG (Ruegen, runnerless, everbearing, and red fruited) were grown in flowerpots and cultivated in photoperiodic conditions of 16 h of light and 8 h of dark at a temperature of 22°C, and a relative humidity of 50%.

### Identification of R2R3-MYB Proteins in Woodland Strawberry

All the protein sequences of *F. vesca* were downloaded from GDR database^1^ and the genome database version 4.0.2a was used. An HMM search with the MYB DNA-binding domain HMM profile (PF000249) was used to blast against strawberry protein database and the *e*-value was set as 1.0 × 10^––3^. Because of the different splicing models for many genes in version 4.0.2a, a total of 499 *MYB* gene transcripts were obtained as a result. For the different splicing of each *MYB* gene, the longest transcript was preserved and the remaining was removed, resulting in 242 *MYB* transcripts obtained. To confirm the presence of MYB DNA-binding domain, the protein sequences of the 242 *MYB* transcripts were used as queries to search against Pfam^2^, SMART^3^, and HMMER^4^ database, the *e*-value was set as 1.0 × 10^––5^. In the end, a total of 118 MYB protein sequences containing more than two MYB DNA-binding domains were obtained. The isoelectric points and molecular weights of the corresponding MYB proteins were obtained from the online database of ExPASY^5^.

### Phylogenetic Analysis

The 125 *R2R3-MYB* genes identified in *Arabidopsis* were obtained from a previous study (Dubos et al., 2010). The MYB protein sequences were downloaded from The *Arabidopsis* Information Resource (TAIR)^6^. The sequences of MYB proteins from *Arabidopsis* and strawberry were aligned using ClustalW (Thompson et al., 1994), and the BLOSUM matrix was used. The parameters of gap opening and extension penalties were 25 and 1, respectively, and the other parameters were default. The phylogenetic tree was constructed using the Neighbour Joining Tree Method in Mega5 (Tamura et al., 2011). The bootstrap was 1,000 replicates. Evolutionary distances were computed with P-distance method and the positions containing gaps and missing data were eliminated with partial deletion option (95%). For the MYB and bHLH proteins, and other corresponding proteins from different species, the phylogenetic tree was constructed as above.

### Yeast Two-Hybrid Assay

Full CDS of *bHLH* and *MYB* genes were amplified and then inserted into BD vector pLexA/pGBKT7 and AD vector pB42AD/pGADT7, respectively, between EcoR I and Xho I using ClonExpress II One Step Cloning Kit (Vazyme Biotech Co., Ltd.). All sequences of the recombinant plasmids were verified by sequencing (Sangon Biotech Co., Ltd. at Shanghai). The LexA yeast two-hybrid was performed as described previously (Xu et al., 2019). For the GAL4 yeast two-hybrid assay, the bait and prey vectors were co-transformed into AH109 yeast cells as described by Lu et al. (2015). The primers used were listed in Supplementary Table 1.

### Yeast One-Hybrid Assay

The 2.0 kb upstream sequences before the ATG of *FvCHS2* and *FvDFR2* genes were amplified from woodland strawberry of RG and inserted into the reporter vector pLacZ using homologous recombination method as above. The recombinant effector plasmids with inserted *MYB* or *bHLH* genes and the reporter plasmids were co-transformed into EGY48 yeast cells and grown at 30°C for 3 days. Then the DNA-binding activity were detected as described in the previous study (Li et al., 2020). To investigate whether bHLH can affect the bioactivity of MYB protein, the cDNA encoding the full-length *bHLH* was inserted into a modified pLexA vector, which lacks the BD domain. The two effector plasmids harboring *bHLH* and *MYB* genes, respectively, were co-expressed with the reporter plasmid in EGY48 yeast cells. Transformed colonies were selected on SD-Trp-His-Ura medium plus X-gal as described previously (Li et al., 2020). The sequences of the CHS2 and DFR2 promoters were listed in Supplementary Table 2 and the potential *cis*-elements of the CHS2 and DFR2 promoters for MBW binding were analyzed by PlantCARE^7^ (Lescot et al., 2002). The primers used were listed in Supplementary Table 1.

### Quantitative RT-PCR Analysis

Strawberry fruits at green stage (about 15 days post anthesis, DPA), white stage (about 22 DPA), pre-turning stage (white receptacles with red achenes), turning stage (2 or 3 days after the pre-turning stage), and ripen stage (3 or 4 days after the turning stage) were harvested. The achenes were removed from all the fruits. and then the total RNA was extracted following the manufacturer’s instructions of RNA Plant Plus Reagent (Tiangen, Beijing, China, Cat.DP437). DNase I was used to remove the genomic DNA contamination. First-strand cDNA was synthesized using EasyScript^®^ One-Step gDNA Removal and cDNA Synthesis SuperMix (Transgen, China). Real-time qPCR was performed using the CFX96 Touch^TM^ Real-Time PCR System (Bio-Rad, United States) with 2 × M5 HiPer SYBR Premix EsTaq (with Tli RNaseH) (Meibio, China, Cat. MF787-01). The qPCR reaction was performed by pre-denaturing at 95°C for 1 min, followed by 40 cycles of denaturing at 95°C for 5 s, annealing at 60°C for 15 s, and extension at 72°C for 15 s. Melting curve detection from 65 to 95°C was done after the cyclic reactions. Transcript abundance of target genes were calculated using 2^–Δ*Ct*^ method in comparison with the internal control gene, *Fv26S* or *FvActin*. Three biological replicates were used in each assay. The experiments were repeated at least three times. The primer sequences were listed in Supplementary Table 3.

### BiFC Assay

The vectors used to produce constructs for the BiFC assays were pXY104 and pXY106, which carry fragments encoding the C- and N-terminal halves of YFP (cYFP and nYFP), respectively. The full-length cDNAs of *MYB* and *bHLH* genes were cloned into pXY104 and/or pXY106 vector to generate corresponding recombinant plasmids. All constructs were transformed into *Agrobacterium tumefaciens* strain GV3101 and infiltrated into tobacco (*Nicotiana benthamiana*) epidermal leaves in the given combinations as described previously (Lu et al., 2015; Xu et al., 2016). After incubation in the dark for 36–40 h, the tobacco leaf samples were examined by confocal microscopy (Leica TCS SP5II) to detect the expression of various fluorescent proteins. The intensity of the laser was set at 30%, the wavelength at 514 nm, and the voltage at 700 v. All the primers used were listed in Supplementary Table 1.

### Luciferase Reporter Assay

The pGreen0800*-Pro_*DFR*2_:Luc* vector was described previously (Li et al., 2020). The full length CDS of *MYB* and *bHLH* genes including *FvMYB22*, *FvMYB64*, *FvMYB105*, and *FvbHLH33* were cloned into pGreen62SK vector and served as effectors. The reporter strain GV3101 harboring *Pro_*DFR*2_:Luc* was mixed with each of the effector strain harboring pGreen62SK, pGreen62SK*-bHLH33* or one of the pGreen62SK-*FvMYB* plasmids (pGreen62SK-*FvMYB22*, pGreen62SK-*FvMYB64*, or pGreen62SK-*FvMYB105*) individually, as well as combinations of plasmids indicated. The mixed culture of each combination was infiltrated into tobacco leaves as described in the results of this article. After incubation in the dark for 36–40 h, the *N. benthamiana* leaves were treated with 1 mM l-luciferin sodium salt (Yeasen, Shanghai, China, Cat. 40903ES02) and kept in the dark for an additional 10 min before the bioluminescence signal were imaged and the intensity measured by a luminescent imaging workstation (5200; Tanon, Shanghai, China). The primers used for luciferase reporter assay are listed in Supplementary Table 1.

### Detection of Anthocyanin and Proanthocyanidin

The method of anthocyanin detection was described in previous studies (Xu et al., 2018; Li et al., 2020). The method of proanthocyanidins detection was described in previous study (Li et al., 2018). DMACA, (−)-epicatechin and 4-hydroxy-3-methoxybenzaldehyde used in the experiments were from Bide Pharmatech. Ltd. (Shanghai, China).

### Transient Overexpression in Strawberry Fruit

The construct of pGreen62SK, pGreen62SK*-bHLH33* or one of the pGreen62SK-*FvMYB* plasmids (pGreen62SK-*FvMYB22*, pGreen62SK-*FvMYB64*, or pGreen62SK-*FvMYB105*) was used for transient overexpression. The method of injection was described in previous study (Zhang et al., 2020). The octoploid strawberry of “HongYan” was used for transient overexpression. The fruit were collected at 7 days after transfection and the measurement of proanthocyanidins was described in the same previous study (Xu et al., 2018). The expression of *FvbHLH33*, *FvMYB22*, *FvMYB64*, *FvMYB105*, *FvCHS2*, and *FvDFR2* was confirmed by qRT-PCR assay.

## Results

### Genome Wide Identification of *R2R3-MYB* Genes in *Fragaria vesca*

In order to identify *R2R3-MYB* genes in strawberry, the HMM profile (Pfam:00249) of MYB DNA-binding domain was used to blast against the woodland strawberry genome database (*F. vesca* Whole Genome v4.0.a2) from GDR (see Text Footnote 1). The blast search resulted in a total of 242 *MYB* genes after removing repetitive redundant sequences. The protein products of 118 *MYB* genes out of the 242 were verified to contain conserved MYB DNA-binding domains through searching Pfam, SMART and HMMER databases. All 118 MYB gene products contain more than two MYB repeat units. Among them, one contains four MYB repeat units, five contain three MYB repeat units. The remaining 112 contain two MYB repeat units, which are R2R3-MYBs (Supplementary Table 4). Through gene sequence alignment analysis, we found that FvH4_1G22020 shares the same sequence with *FvMYB10*, thus annotated as *FvMYB10*. Blast results showed that the sequences of FvH4_2g31100 and FvH4_6g34650 were almost consistent with *FaMYB9* and *FaMYB11*, respectively. We therefore named FvH4_2g31100 and FvH4_6g34650 as *FvMYB9* and *FvMYB11*. The remaining *R2R3-MYB* genes were named according to the order of their chromosomal locations in the strawberry genome browser (Supplementary Table 4). The physical and chemical property prediction analysis was performed for the 112 R2R3-MYB proteins using ExPASy database. The results showed that the sizes of these MYB proteins are significantly different. For example, the protein product of FvH4_7g19850 is composed of 1703 amino acids, while other MYB proteins are much smaller, containing amino acids of a number ranging from 127 to 562. The isoelectric points of MYB proteins have been predicted to range from 4.82 to 10.10, and the presumed molecular weights are in a range of 15–62.7 kD, except for the protein product of FvH4_7g19850, the molecular weight of which is 185 kD (Supplementary Table 4).

### Identification of Potential MYB and bHLH Proteins Involved in Regulating Flavonoids Biosynthesis

**FIGURE 1:**
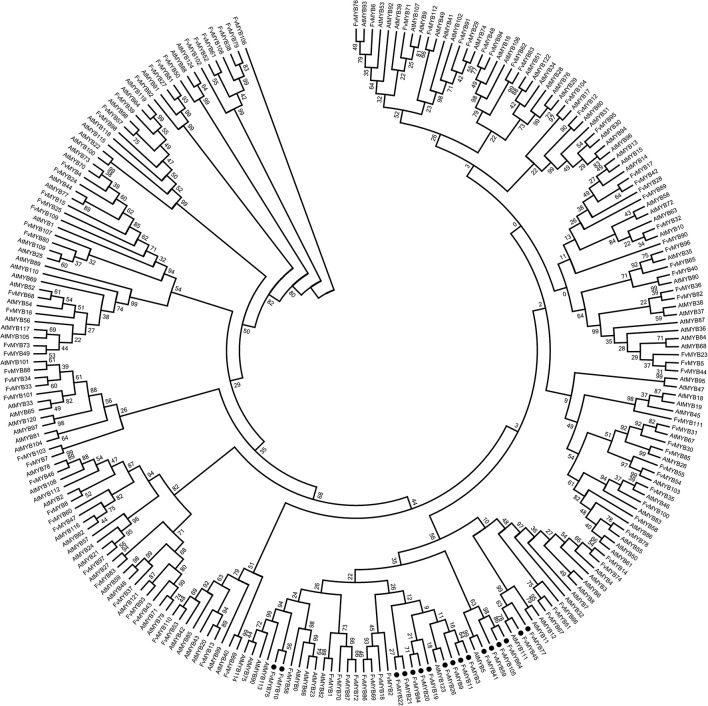
Phylogenetic analysis of R2R3-MYB proteins of *Arabidopsis* and woodland strawberry. The numbers above the line are the bootstrap values. Black solid circles indicate those related to flavonoid biosynthesis.

To identify which *R2R3-MYB* genes in strawberry are possibly involved in flavonoids biosynthesis, the sequences of the 112 strawberry *R2R3-MYB* genes identified by us and the 125 *R2R3-MYB* genes from *Arabidopsis* were phylogenetically analyzed. The results showed that a total of 17 strawberry *MYB* genes clustered with *AtMYB*s that are involved in flavonoids biosynthesis (highlighted in black solid circles in Figure 1). We also included *R2R3-MYB* genes from other species with known functions in flavonoids synthesis for comparison, including those from grape, apple, cotton, tomato, maize and freesia. These *R2R3-MYB* genes were also phylogenetically analyzed together with the 17 strawberry *MYB* genes. Taken together,12 strawberry *R2R3-MYB* genes were identified as potentially involved in flavonoids biosynthesis (highlighted in black solid circles, Supplementary Figure 1). Specifically, *FvMYB10* and *FvMYB75* clustered with *MdMYB10* and *MdMYB1*, which promote anthocyanin biosynthesis (Espley et al., 2007). *FvMYB3*, *FvMYB9*, and *FvMYB11* clustered with *FaMYB9* and *FaMYB11*, which were found to complement the phenotype of transparent testa in *Arabidopsis tt2-1* mutant (Schaart et al., 2013), suggesting a function of regulating proanthocyanidins biosynthesis. *FvMYB64* and *FvMYB105* were homologous to *VvMYBPA1*, which is specific to the regulation of proanthocyanidins biosynthesis in seeds (Bogs et al., 2007). *FvMYB45* and *FvMYB77* were similar to maize *P* (Grotewold et al., 1994) and *AtMYB111* (Stracke et al., 2007), respectively, suggesting that both of them might be related to flavonols synthesis. *FvMYB41* clustered with *VvMYB5b*, which contributes to the regulation of anthocyanin and proanthocyanidins biosynthesis in the developing grape fruits (Deluc et al., 2008). *AtMYB123* did not explicitly cluster with any known genes, but when *AtMYB123* was used as a query to blast against strawberry genome, *FvMYB21* and *FvMYB22* was also among the results, displaying high similarity to *AtMYB123*, suggesting that *FvMYB21* and *FvMYB22* are potentially related to proanthocyanidins biosynthesis.

To screen bHLH components of MBW complexes, we used three *Arabidopsis* bHLH proteins as queries to perform BLASTP searches against *F. vesca* protein database. The three *Arabidopsis* bHLH proteins, including AtTT8, AtEGL3, and AtGL3, have been shown to have partially overlapping expression patterns and redundant functions in flavonoids biosynthesis. By the searches, we found that no matter which one of AtTT8, AtEGL3, and AtGL3 was used for blasting, the matching proteins with higher similarity were always the products of FvH4_2g23700, FvH4_7g14230, and FvH4_5g02520 (Supplementary Table 5). A phylogenetic analysis of these three strawberry bHLH proteins and those from other 16 plants was performed, and we found that FvH4_2g23700, FvH4_7g14230, and FvH4_5g02520 clustered with FabHLH3, FabHLH33 and FaMYC1 of *Fragaria* × *ananassa*, respectively (Supplementary Figure 2). Consequently, the bHLH proteins from woodland strawberry were named as FvbHLH3, FvbHLH33, and FvMYC1, and considered potential components of MBW complexes for flavonoids biosynthesis.

### The Expression Pattern of *R2R3-MYB* and *bHLH* Genes Correlates With Anthocyanin and Proanthocyanidin Accumulation in Strawberry Fruits

**FIGURE 2:**
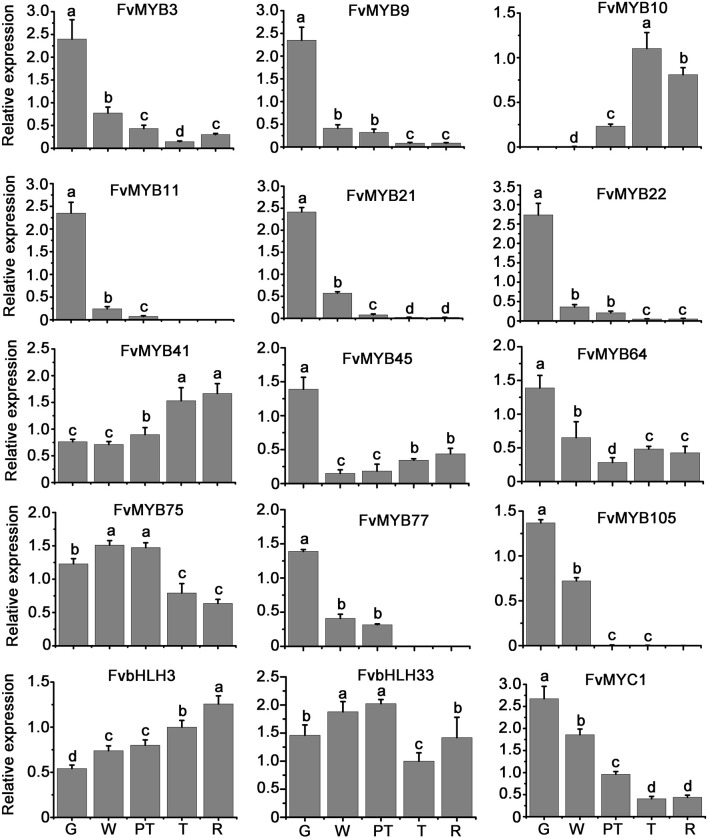
Relative expression levels of *MYB* and *bHLH* genes in fruits of different development stages. Values were normalized to the expression level of an internal control, *Fv26S*. Data are presented as the mean of biological replicates ± SD (*n* = 3). G, green stage; W, white stage; PT, pre-turning stage; T, turning stage; R, ripe stage. The letters a–d indicate statistically significant differences, as determined by Tukey’s LSD test (*P* ≤ 0.05).

**FIGURE 3:**
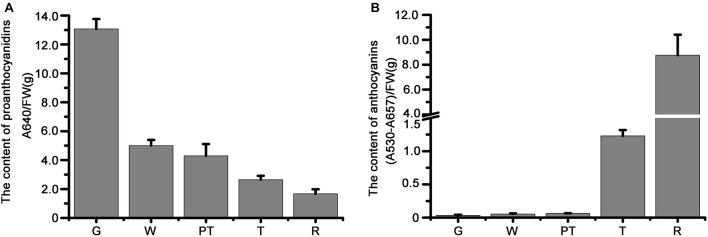
The measurement of anthocyanins and proanthocyanidins in fruits of different development stages. **(A)** Proanthocyanidins; **(B)** anthocyanins. G, green stage; W, white stage; PT, pre-turning stage; T, turning stage; R, ripe stage.

We studied the transcript levels of *MYBs* and *bHLHs*, and their effects on the accumulation of flavonoids in strawberry fruits at five stages of fruit development, including green stage, white stage, pre-turning stage, turning stage and ripe stage. qRT-PCR results showed that the transcript level of a *bHLH* gene (*FvMYC1*) and nine *MYB* genes (*FvMYB3*, *FvMYB9*, *FvMYB11*, *FvMYB21*, *FvMYB22*, *FvMYB45*, *FvMYB64*, *FvMYB77*, and *FvMYB105*) was higher at green stage, but significantly decreased at the other four stages (Figure 2 and Supplementary Figure 3), matching proanthocyanidins biosynthesis pattern, which accumulated mainly at the early stages, especially at the green stage (Figure 3A). Specifically, the expression of *FvMYB11*, *FvMYB21*, *FvMYB77*, and *FvMYB105* were not detected during turning stage and ripe stage. By contrast, the expression of *FvMYB10* was extremely low in green and white stages, but significantly increased during the late development stages, especially the turning stage, resembling anthocyanin biosynthesis pattern, which accumulated in a large amount during turning and maturation stages (Figure 3B). The expression of *FvMYB41* and *FvbHLH3* gradually increased accompanying the development of fruit and peaked at ripe stage (Figure 2 and Supplementary Figure 3), also aligned with anthocyanins accumulation (Figure 3B). The expression of *FvMYB75* and *FvbHLH33*, however, was detected at all five stages, with higher expression level at early stages from green to pre-turning than turning and ripe stages (Figure 2 and Supplementary Figure 3). Taken together, the *bHLH* gene *FvMYC1*, and the nine *MYB* genes including *FvMYB3*, *FvMYB9*, *FvMYB11*, *FvMYB21*, *FvMYB22*, *FvMYB45*, *FvMYB64*, *FvMYB77*, and *FvMYB105*, might be involved in proanthocyanidins biosynthesis at early stages of fruit development. *FvMYB10*, *FvMYB41*, and *FvbHLH3* might be associated with the accumulation of anthocyanins at later stages. The proposed functions of woodland strawberry *MYB* and *bHLH* genes are consistent with the phylogenetical analysis (Supplementary Figure 1).

### Interaction Between the Putative Components of Woodland Strawberry MBW Complexes *in vitro* and *in vivo*

**FIGURE 4:**
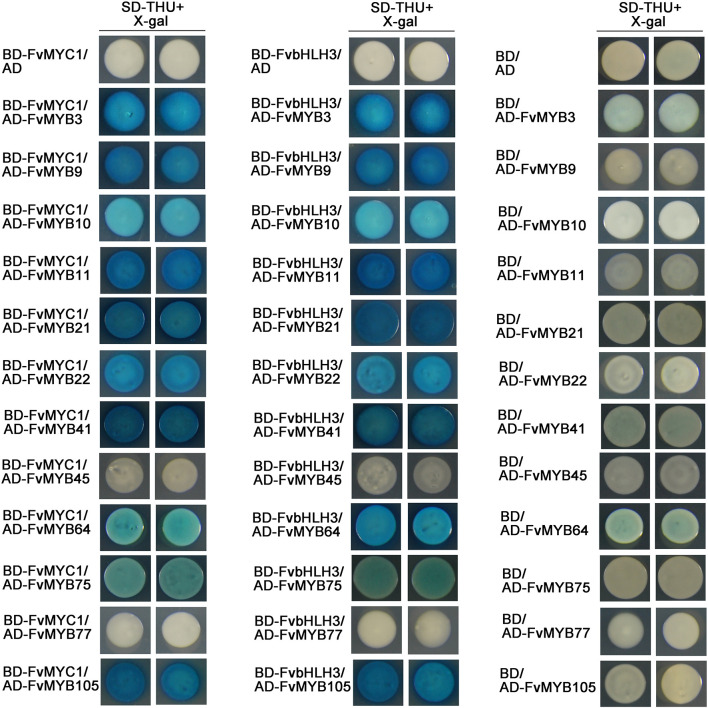
The interaction of MYB and bHLH proteins in yeast cells. LexA yeast two-hybrid assays showing interactions between FvMYB proteins and FvbHLH proteins. Yeast cells were grown on selective medium (SD-Trp, -His, -Ura; SD-THU) with 80 mg/L *X*-gal. Blue precipitates represent cumulative β-galactosidase activity resulting from the activation of the *LacZ* reporter gene by interacting proteins. Two representative colonies were shown for each interaction.

To study whether these potential MYB and bHLH proteins can form MBW complexes, we first investigated the interaction among them *in vitro* using LexA yeast two-hybrid system. When FvbHLH3 or FvMYC1 were used as a bait (fused with DNA-binding domain, BD), they both could interact with every MYB protein tested (fused with Activation domain, AD) except for FvMYB45 or FvMYB77 (Figure 4). FvbHLH33, however, displayed strong self-activation when fused to LexA BD as a bait. This is also true when only the N-terminal fragment of FvbHLH33 was used (Supplementary Figure 4). Considering that 3-AT can be used to inhibit self-activation in GAL4 yeast two-hybrid system, the interaction between FvbHLH33 and MYB proteins were studied in GAL4 system instead. 20 mM 3-AT was introduced to inhibit self-activation using FvbHLH33 as a bait, and we were able to detect interactions between FvbHLH33 and 10 individual MYB proteins, including FvMYB3, FvMYB9, FvMYB10, FvMYB11, FvMYB21, FvMYB22, FvMYB41, FvMYB75, FvMYB77, and FvMYB105, but not FvMYB45 or FvMYB64 (Supplementary Figure 5).

**FIGURE 5:**
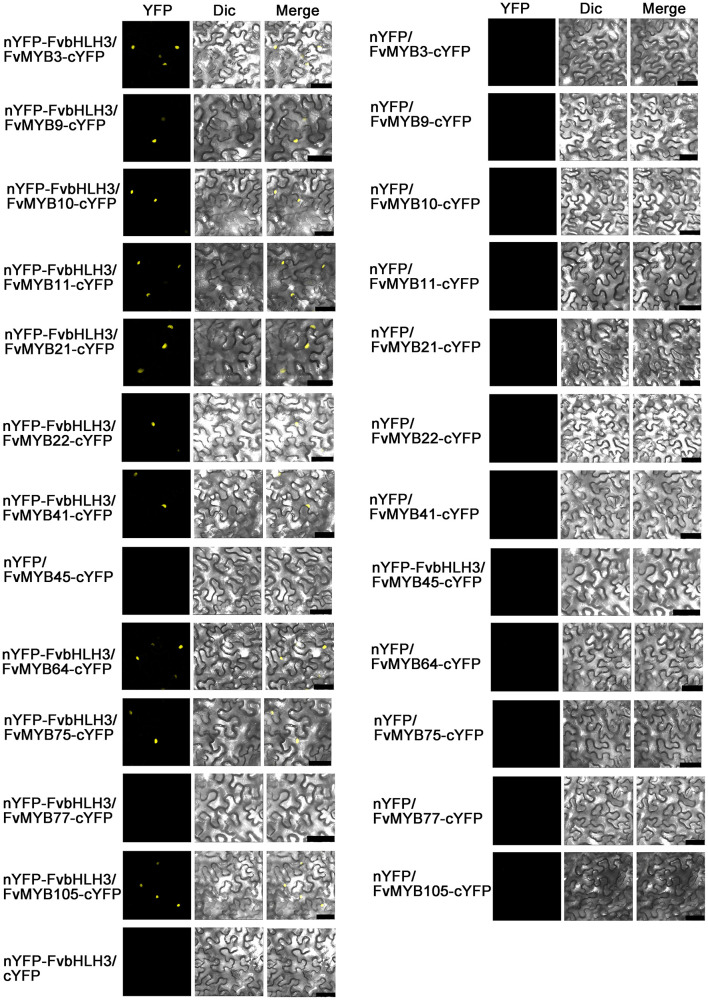
BiFC assay showing interactions between FvMYB proteins and FvbHLH proteins in tobacco cells. Combinations of constructs encoding the indicated proteins were co-transformed into tobacco leaf epidermal cells. Overlays of fluorescence and bright field were shown. Dic, differential interference contrast. Bars, 50 μm.

We also performed BiFC experiments to furtherly investigate the interactions between bHLH and MYB proteins *in vivo*. As shown in Figure 5, when MYBs or FvbHLH3 were expressed individually, no over-lapping fluorescent signal of YFP was seen. When FvbHLH3 and any of the 10 MYB proteins, including FvMYB3, FvMYB9, FvMYB10, FvMYB11, FvMYB21, FvMYB22, FvMYB41, FvMYB64, FvMYB75, and FvMYB105, were co-expressed in tobacco cells, strong fluorescent signal of YFP was observed. However, when FvbHLH3 and FvMYB45 or FvMYB77 were co-expressed, no YFP signal was detected. Similarly, FvMYC1 or FvbHLH33 was observed to interact with the same 10 MYB proteins, but not FvMYB45 or FvMYB77 (Supplementary Figure 6). The interaction between FvMYB64 and FvbHLH33 was not detected in yeast two-hybrid, but the interaction was detected in BiFC assay, likely due to the difference between the *in vitro* and *in vivo* experimental systems. Taken together, the results *in vitro* and *in vivo* suggested that the 10 MYB proteins, except for FvMYB45 and FvMYB77, could possibly form functional MBW complexes with FvbHLH3, FvbHLH33, or FvMYC1.

### Most MYB Proteins Bind to the Promoters of Key Enzyme Genes in Flavonoids Biosynthesis

**FIGURE 6:**
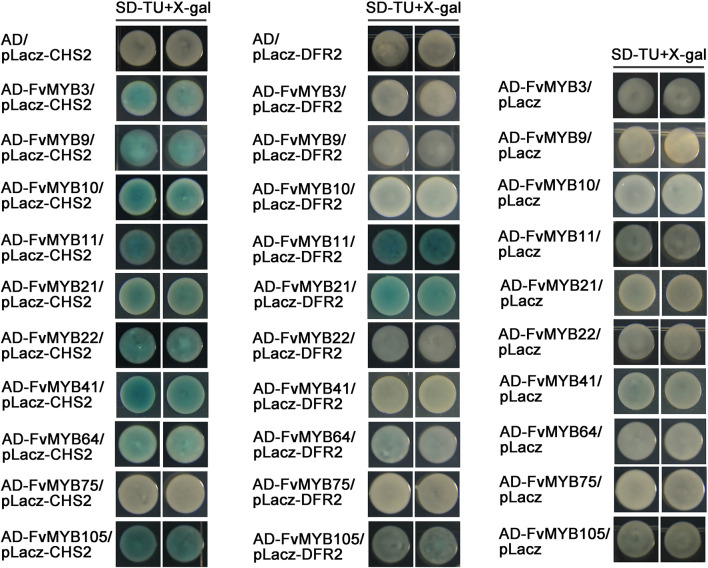
Yeast one-hybrid assays showing the binding of MYB proteins to the promoters of *CHS2* and *DFR2*. Yeast cells were grown on selective medium (SD-Trp, -Ura; SD-TU) with 80 mg/L *X*-gal. Blue precipitates represent cumulative β-galactosidase activity resulting from the activation of the LacZ reporter gene by binding. Two representative colonies were shown for each binding.

In a MBW complex, MYB protein is the decisive factor for the regulation of specific flavonoids synthesis genes. To confirm whether the identified MYB proteins have a potential role in regulating genes involved in flavonoids biosynthesis, we first analyzed whether the promoters of CHS2 and DFR2 contained *cis*-elements bound by MBW complexes. As shown in Supplementary Figure 7, the promoter regions of CHS2 and DFR2 contain binding elements of MYB proteins. Then, we tested in yeast one-hybrid system to see whether these MYB proteins are able to bind to the promoters of *CHS2* and *DFR2* genes, which encode key enzymes related to the flavonoids synthesis pathways. As shown in Figure 6, all tested MYB proteins, except for FvMYB75, displayed binding activity to the promoter of *CHS2* gene. However, only FvMYB11 and FvMYB21 bound to the *DFR2* promoter.

### The Heterodimers Formed by MYBs and bHLHs Activate the Expression From DFR2 Promoter

**FIGURE 7:**
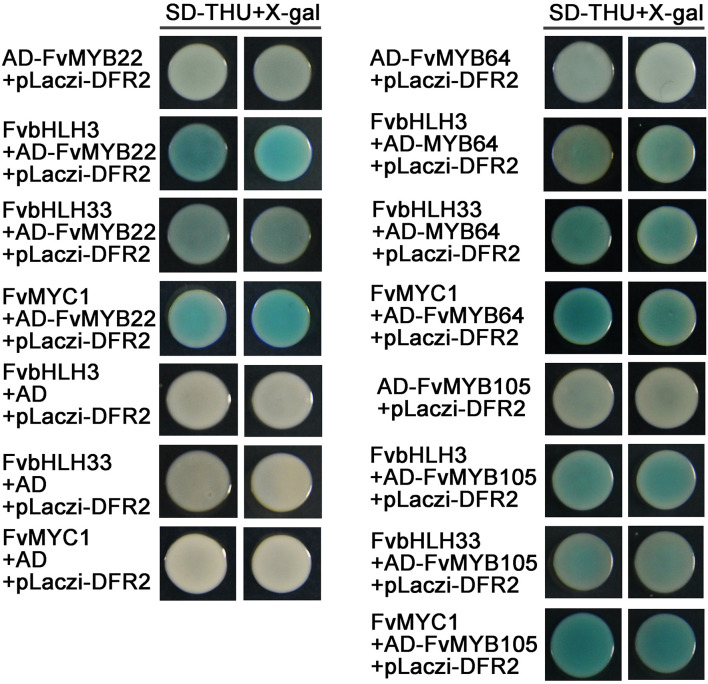
The effect of interactions between MYB and bHLH proteins on the *DFR2* promoter fused with *LacZ* reporter gene. Yeast cells were grown on selective medium (SD-Trp, -His, -Ura; SD-THU) with 80 mg/L *X*-gal. Blue precipitates represent cumulative β-galactosidase activity resulting from the activation of the *LacZ* reporter gene by binding. Two representative colonies were shown for each binding.

We observed that most of the MYB proteins (except for FvMYB11 and FvMYB21) in strawberry could not bind to the promotor of *DFR2* in yeast one-hybrid assay (Figure 6), nor the three bHLH proteins (FvbHLH3, FvbHLH33, or FvMYC1) (Supplementary Figure 8). To explore whether bHLH proteins are in need for those MYBs to bind to the promotor of *DFR2*, we test the DNA-binding activity of heterodimers formed between five FvMYB proteins (FvMYB10, FvMYB22, FvMYB64, FvMYB75, and FvMYB105) and three FvbHLH proteins (FvbHLH3, FvbHLH33, or FvMYC1) in yeast one-hybrid assay. As shown in Figure 7, transformation of yeast cells with either one of the five MYB proteins fused to AD did not show positive *DFR2* promotor binding activity. However, when either one of the three bHLH proteins, FvbHLH3, FvbHLH33 or FvMYC1, was co-expressed with AD-FvMYB22, AD-FvMYB64, or AD-FvMYB105, the report gene of *LacZ* was activated and the yeast cells displayed blue on media containing *X*-gal, demonstrating the binding of MYB proteins to the promotor of *DFR2* in the presence of bHLH proteins. For FvMYB75 and FvMYB10, the reporter gene *LacZ* can only be activated in the presence of FvMYC1, but not FvbHLH3 and FvbHLH33 (Supplementary Figure 9), suggesting a requirement of specific bHLH protein for the DNA-binding activity of FvMYB75 or FvMYB10.

**FIGURE 8:**
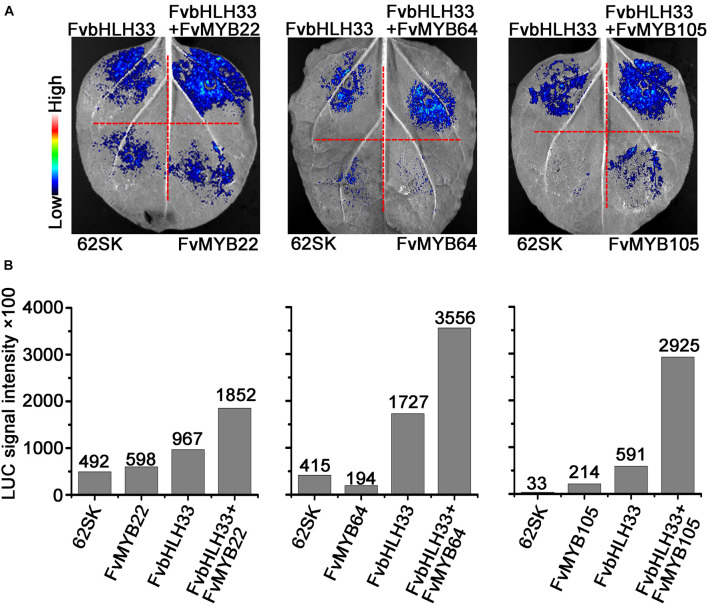
The effect of interactions between MYB and bHLH proteins on the DFR2 promoter fused with luciferase reporter gene. The constructs encoding the indicated combinations of proteins were co-transformed into tobacco leaf epidermal cells, and the luminescence images were captured using a CCD imaging system **(A)**, the intensity of luminescence was indicated in **(B)**.

To further confirm the requirement of FvbHLH proteins for the DNA-Binding activity of FvMYB proteins *in vivo*, we transiently expressed FvbHLH33 and three FvMYB proteins (FvMYB22, FvMYB64, and FvMYB105) separately, or as a combination of FvbHLH33 with one of the three FvMYB proteins in *N. benthamiana* leaves, along with a reporter luciferase gene under the control of *DFR2* promoter. As shown in Figures 8A,B, when FvMYB22, FvMYB64, or FvMYB105 was expressed alone, the reporter gene was not significantly induced, whereas these MYB proteins induced the reporter gene in the presence of FvbHLH33, leading to a strong luminescence signal when the substrate of luciferin was added. These results, together with results from yeast one-hybrid suggested that the heterodimers between FvMYBs and FvbHLHs were necessary for activating some flavonoids synthesis genes.

### Co-expression of FvMYB and FvbHLH Proteins Promotes the Accumulation of Proanthocyanidins

**FIGURE 9:**
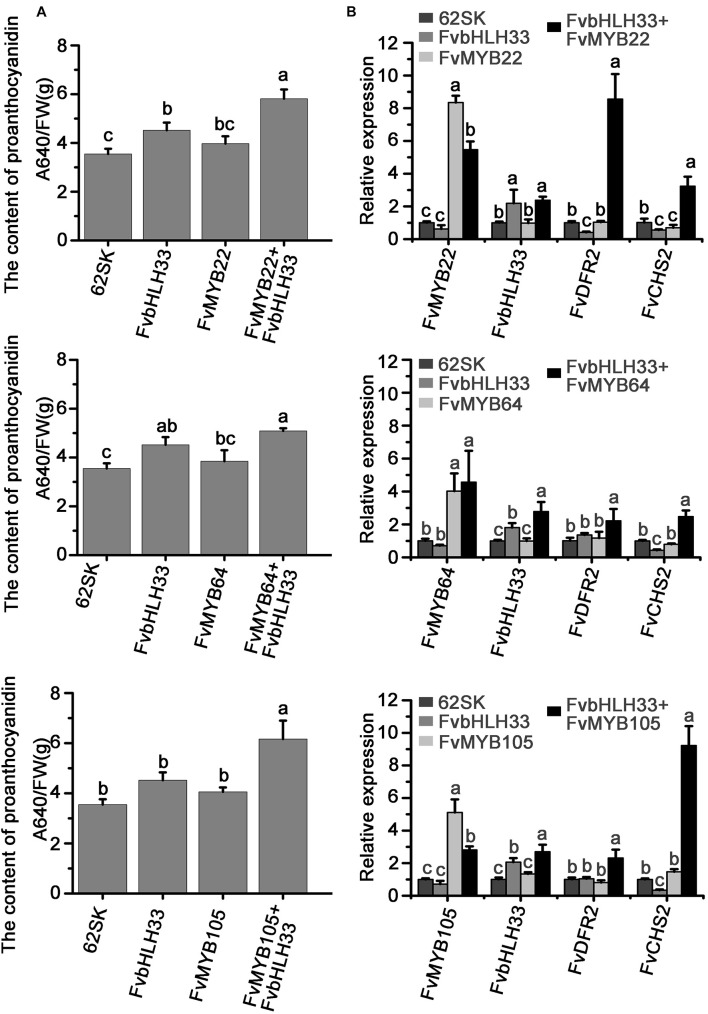
The complex of FvMYB and FvbHLH33 can promote the accumulation of proanthocyanidin in strawberry fruits. The *Agrobacterium* containing the indicated combinations of constructs were injected into strawberry fruits and the content of proanthocyanidins was detected at 7 days after injection. **(A)** Measurement of proanthocyanidin related to the transient expression of FvMYB and FvbHLH proteins. **(B)** Relative expression levels of *FvMYBs, FvbHLH33*, *FvCHS2*, and *FvDFR2* genes from transient expression. *Fv26S* served as an internal control. Data are presented as the mean ± SD (*n* = 3). The letters a–c indicate statistically significant differences, as determined by Tukey’s LSD test (*P* ≤ 0.05).

To confirm the biofunction of strawberry MBW complexes in flavonoids synthesis, we co-expressed FvMYB and FvbHLH proteins in the fruits and measured the flavonoids accumulation. FvMYB22, FvMYB64, and FvMYB105 are homologs of MYB proteins in other plants that regulate proanthocyanidin synthesis (Supplementary Figure 1) and mainly expressed at green stage (Figure 2 and Supplementary Figure 3). FvbHLH33 has been shown to interact with all three FvMYB proteins in yeast and tobacco cells. We transiently expressed FvbHLH33 and the three FvMYB proteins individually, or a combination of FvbHLH33 and one of the three FvMYB proteins in green stage strawberry fruit. The accumulation of proanthocyanidins was measured following the expression. As shown in Figures 9A,B and Supplementary Figure 10, the accumulation of procyanidins did not significantly increase following the expression of either FvbHLH33 or any of the three FvMYB proteins alone. However, the expression levels of flavonoid biosynthesis related genes CHS2 and DFR2 was significantly enhanced and the accumulation of proanthocyanidins was significantly increased in strawberry fruits when FvbHLH33 and one of the three FvMYB proteins were co-expressed (Figures 9A,B and Supplementary Figure 10). These results confirmed the function of the identified MBW components in strawberry flavonoids biosynthesis.

## Discussion

In this study, we identified 112 *R2R3-MYB* genes in woodland strawberry by using HMM profile of MYB DNA-binding domain to blast strawberry genome database and further verified the presence of the MYB DNA-binding domain in Pfam, SMART and HHMER (Supplementary Table 4). Recently, Liu et al. (2019) have identified *R2R3-MYB* genes in six Rosaceae species including strawberry (*F. vesca*), but they identified 105 *R2R3-MYB* genes which was seven genes fewer than what we found. The difference was likely because they used the 125 R2R3-MYB from *Arabidopsis* to perform repetitive BLASTP search against the strawberry genome version 1.1.a2, in which there were still many scaffolds (Darwish et al., 2015). In our study, we used HMM profile of MYB DNA-binding domain to search against the strawberry genome version 4.0.a2, which contains 34,007 genes, 511 more genes than the 33,496 genes in version 1.1.a2 (Li et al., 2019). We speculate that some *R2R3-MYB* genes might have been omitted or wrongly identified due to the annotation errors in the version 1.1.a2. For example, mrna32181, mrna16655, mrna01311 and mrna28755 were identified as *R2R3-MYB* genes in version 1.1.a2 by Li et al. (2019). When using these genes to blast against version 4.0.a2, each of these four genes was annotated as two or three different genes in version 4.0.a2 (Supplementary Figure 11), indicating that there were wrong annotations in version 1.1.2a. Given that genomic information in version 4.0 is at chromosomal level, 112 *R2R3-MYB* identified in our study have higher confidence levels.

It was proposed that in a MBW complex, MYB protein is the decisive factor for the regulation of specific flavonoids synthesis (Baudry et al., 2004). In our study, through phylogenetic tree analysis of *R2R3-MYB* genes from different plants including *Arabidopsis*, grape, pear, strawberry, etc., we obtained 12 potential *MYB* genes that regulate flavonoids synthesis in strawberry. Among these 12 *MYB* genes, *FvMYB10* and *FvMYB75* clustered with *MdMYB1* and *MdMYB10* (Supplementary Figure 1), which were known to promote anthocyanin accumulation in apple (Espley et al., 2007; Gonzalez et al., 2008). It has been reported that over-expression of *FvMYB10* or *FaMYB10* (homolog of *FvMYB10* in cultivated strawberry *Fragaria* × *ananassa*) resulted in elevated concentrations of anthocyanin in root, leaves, petioles, stigmas, petals and fruits (Lin-Wang et al., 2014; Medina-Puche et al., 2014; Wang et al., 2020). Additionally, Hawkins et al. (2016) identified an SNP (a specific SNP mutation of G to C, leading to W12S variant) in *FvMYB10* resulting in abolished accumulation of anthocyanins in the fruits of the woodland strawberry Yellow Wonder (YW). Furthermore, in the yellow and red-fruited varieties tested, the C (W12S) in FvMYB10 is 100% associated with the yellow color of the fruit, while the G is 100% associated with the red color (Hawkins et al., 2016; Zhang et al., 2017). Interestingly in YW, there is no accumulation of anthocyanins in fruits, but the accumulation is normal in petioles (Xu et al., 2014). In addition, two *Fragaria pentaphylla* subvarieties displayed red and white skin color, the relatively low expression level of *FpMYB10* might have contributed to the white fruit variety. The achenes of both varieties, however, are red (Bai et al., 2019). These results suggested that other factors besides FvMYB10 or FpMYB10 might be involved in the regulation of anthocyanin accumulation. In our study, the expression of *FvMYB41* displayed expression pattern similar to *FvMYB10* during the five development stages (Figure 2 and Supplementary Figure 3), resembling anthocyanin biosynthesis pattern (Figure 3B), suggesting that FvMYB41 might have a role in promoting anthocyanin accumulation. Through the phylogenetic tree analysis, we found that *FvMYB41* was most genetically related to *VvMYB5b* from grape and *FhMYB5* from *Freesia*, both of which have been proven to promote the accumulation of anthocyanin and proanthocyanidins (Deluc et al., 2008; Li et al., 2018). By comparing the sequences of *VvMYB5b*, *FhMYB5*, and *FvMYB41*, we found that the similarity of all three was greater than 45% (Supplementary Table 6). The most conserved sequences were within the DNA-binding R2R3 domain as well as the C1 and C3 motifs in the C-terminal regions of the three genes (Supplementary Figure 12). Thus, it was speculated that FvMYB41 likely serves as a homologous protein of VvMYB5b and FhMYB5 in strawberry that promotes flavonoids biosynthesis. However, this notion needs to be tested in future study.

It has been known that MBW complexes composed of MYB-bHLH-WD40 play key roles in regulating flavonoids biosynthesis in plants. In this study, we confirmed the interactions between potential components of MBW in woodland strawberry. We detected the interaction between twelve MYB proteins (FvMYB3, FvMYB9, FvMYB10, FvMYB11, FvMYB21, FvMYB22, FvMYB41, FvMYB45, FvMYB64, FvMYB75, FvMYB77, and FvMYB105) and three bHLH proteins (FvbHLH3, FvbHLH33, and FvMYC1). Using the yeast two hybrid and BiFC assays, we found that except for FvMYB45 and FvMYB77, all the other MYBs can interact with either one of FvbHLH3, FvbHLH33, or FvMYC1 (Figures 4, 5 and Supplementary Figures 5, 6). In addition, we found that FvTTG1, a WD40 protein, interacted with all the three bHLH proteins (Supplementary Figure 13). All these results indicated that the strawberry MBW component proteins identified in this study could form MBW complexes. Interestingly, in octoploid strawberry, FabHLH3, FabHLH33, and FaMYC1 can interact with FaTTG1, but only FabHLH3 can interact with FaMYB9, FaMYB11 (Schaart et al., 2013). Whereas, we found that both FvMYB9 and FvMYB11 interacted with FvbHLH33 and FvMYC1 in woodland strawberry (Figure 4 and Supplementary Figure 5). For these paradoxical results between woodland strawberry and octoploid strawberry, it is likely because that Schaart et al. (2013) used the C terminal of FvMYC1 and FvbHLH33 but not the full length of CDS for yeast two-hybrid assays (Supplementary Figures 14, 15). Only the full-length protein of FvbHLH33, but not the C-terminus fragment, interacted with MYB proteins in our assay (Supplementary Figures 5, 16). Even though Schaart et al. (2013) had demonstrated the direct interactions between FabHLH3 and the two FaMYB proteins (FaMYB9 and FaMYB11), they only did so in yeast cells, not in plant cells (Schaart et al., 2013). In our study, we provided evidence to support the interactions between FvMYB proteins and FvbHLH proteins in both yeast (Figure 4 and Supplementary Figure 5) and plant cells (Figure 5 and Supplementary Figure 6).

To further test whether FvMYB proteins or FvbHLH proteins are involved in the regulation of flavonoids biosynthesis-related genes, we examined the DNA-binding activity of FvMYB proteins, as well as the requirement of FvbHLH proteins, using reporter genes (*LacZ* and *Luciferase*) under the control of the promoters of *CHS2* and *DFR2*, two key enzyme genes related to the flavonoids biosynthesis pathways. Most of the FvMYB proteins except for FvMYB75 displayed DNA-binding activity to *CHS2* promoter. However, most of the FvMYB proteins and three bHLH proteins were not able to bind to *DFR2* promoter except for FvMYB11 and FvMYB21 (Figure 6 and Supplementary Figure 8). It has been reported that in *Arabidopsis*, AtTT2 (AtMYB123) can’t bind by itself to the promoter of *BAN*, a gene involved in proanthocyanidins production. AtTT8 (AtbHLH042) must be present for AtTT2 to bind to the *BAN* promoter in yeast (Baudry et al., 2004). Considering that MYB proteins often form complexes with bHLH proteins to regulate flavonoids biosynthesis, we further examined whether co-expression of FvbHLH proteins could promote the binding of FvMYB proteins to the promoter of *DFR2*. Our results showed that co-expression of either one of the three FvbHLH proteins (FvbHLH3, FvbHLH33, and FvMYC1) with FvMYB22, FvMYB64, or FvMYB105 in yeast cells significantly increased the expression of the reporter gene *LacZ* from *DFR2* promotor, suggested that FvbHLHs can promote the binding activity of FvMYBs to the promoter of *DFR2* (Figure 7). The requirement of FvbHLH protein for the gene induction function of FvMYB proteins was also demonstrated in tobacco cells. FvMYB22, FvMYB64, or FvMYB105 induced the expression of luciferase from *DFR2* promoter when FvbHLH33 was co-expressed (Figure 8). This was further confirmed by the accumulation of flavonoids in strawberry fruits. When FvbHLH33 was co-expressed with either one of FvMYB22, FvMYB64, or FvMYB105 in green stage strawberry fruits, the accumulation of proanthocyanidins was significantly increased (Figure 9A). But for FvMYB75 or FvMYB10, only FvMYC1 could help increase their DNA-binding activity, leading to the induction of *LacZ* expression (Supplementary Figure 9). These results suggested that some FvMYB proteins only form complexes with specific FvbHLH proteins. It has been reported that the maize R2R3-MYB protein C1 co-worked with the bHLH protein R to activate the expression of anthocyanin biosynthesis genes (Grotewold et al., 2000). Later studies showed that the dimerization domain of R behaves as a switch that permits distinct configurations of the regulatory complex to be tethered to different promoters of target genes (Feller et al., 2006; Kong et al., 2012). These studies suggested that bHLH protein may determines the target gene of MBW complexes. The biofunctions of the three FvbHLH proteins in woodland strawberry, including FvbHLH3, FvbHLH33, and FvMYC1, remain to be further investigated.

Since FvMYB22, FvMYB64, and FvMYB105 clustered with VvMYBPA1, FaMYB9, and FaMYB11, which were reported to promote proanthocyanidins biosynthesis. We tested whether these three MYBs can bind to the promoter of *FvLAR*, a key enzyme gene regulating the proanthocyanidins synthesis. Our results showed that FvMYB22, FvMYB64, and FvMYB105 bound to *FvLAR* promoters only in the presence of FvMYC1 (Supplementary Figure 17). Considering the interaction with other FvMYB proteins in activating *CHS2* and *DFR2* promoters, FvMYC1 might be a more universal bHLH partner for FvMYB proteins and play more extensive roles in flavonoids biosynthesis than the other two bHLH proteins, FvbHLH3 and FvbHLH33. In fact, even when the transcripts of *FvbHLH33* was significantly reduced in FvbHLH33 RNAi lines, the pigmentation was not significantly altered in those lines when compared with WT (Lin-Wang et al., 2014). This also indicated that other bHLH proteins, especially FvMYC1, might compensate for the lack of FvbHLH33 in this case. Recently, it was found that FvbHLH9 can promote anthocyanin accumulation in strawberry fruits, but FvMYB10 could not promote the expression of downstream target genes when only bHLH9 was present (Li et al., 2020), likely because the lack of WD40 protein. The results of existing studies strongly suggest that there are other regulatory MBW proteins in strawberry that control the synthesis and accumulation of anthocyanins.

## Conclusion

In this study, we identified novel MBW components in woodland strawberry utilizing phylogenetic analysis, and confirmed the biofunctions of several members in flavonoids accumulation using molecular and biochemical methods. This work provided not only the basis for study of flavonoids synthesis in strawberry, but also potential economic benefit in strawberry cultivation. As transcriptional regulators, further studies of MBW complexes and their biological role in other important physiological processes and regulatory mechanism will contribute to better understanding of strawberry biology.

## Data Availability Statement

The datasets presented in this study can be found in online repositories. The names of the repository/repositories and accession number(s) can be found in the article/Supplementary Material.

## Author Contributions

PX and HL designed the experiments. PX, MC, and YL carried out the experiments. PX, MC, and KX collected the data. PX, LW, and HL analyzed the data. PX, LW, CM, YL, and HL wrote the manuscript with input from all other authors. All authors contributed to the article and approved the submitted version.

## Conflict of Interest

The authors declare that the research was conducted in the absence of any commercial or financial relationships that could be construed as a potential conflict of interest.

## Publisher’s Note

All claims expressed in this article are solely those of the authors and do not necessarily represent those of their affiliated organizations, or those of the publisher, the editors and the reviewers. Any product that may be evaluated in this article, or claim that may be made by its manufacturer, is not guaranteed or endorsed by the publisher.

## Footnotes

1
https://www.rosaceae.org/

2
https://www.ebi.ac.uk/Tools/pfa/pfamscan/

3
http://smart.embl-heidelberg.de/

4
https://www.ebi.ac.uk/Tools/hmmer/

5
https://www.expasy.org/

6
http://www.arabidopsis.org/

7
http://bioinformatics.psb.ugent.be/webtools/plantcare/html/
