# Supplementary material for: Identification of MBW Complex Components Implicated in the Biosynthesis of Flavonoids in Woodland Strawberry
Source: Front Plant Sci. 2021 Nov 8;12:774943. doi: 10.3389/fpls.2021.774943 (PMC8606683; doi:10.3389/fpls.2021.774943)
Supplement: Supplementary file 1 [file Data_Sheet_1.docx]

**Supplementary Informations:**

Supplementary Figure 1. Phylogenetic relationships between 17 R2R3-MYB transcription factors from woodland strawberry and 22 R2R3-MYB proteins from other plant species. Phylogenetic analyses were performed using the neighbor-joining method by the MEGA version 5 program. The numbers next to the nodes are bootstrap values from 1,000 replicates. Black solid circles represent that these MYB proteins are related to flavonoid biosynthesis. GenBank accession numbers of R2R3-MYB proteins from other plant species are as follows: AtMYB123 (CAC40021), AtMYB75 (AAG42001), AtMYB90 (AAG42002), AtMYB12 (CAB09172), AtMYB11 (AEE80369), AtMYB111 (AED95797), AtMYB113 (AEE34501), AtMYB114 (ANM58977), AtMYB5 (AAC49311), MdMYB10 (DQ267896), MdMYB1 (NP_001288045), VvMYBA1 (AB242302), VvMYBPA1 (CAJ90831), VvMYB5b (AAX51291), FhMYB5 (MK168337), maize P (U57002), ZmC1 (AF292540), FaMYB9 (JQ989281), FaMYB11 (JQ989282), Petunia AN2 (EF423868), SlANT1 (AY348870), GhMYB10 (AJ554700).

Supplementary Figure 2. Phylogenetic relationships between 3 bHLH transcription factors from woodland strawberry and 16 bHLH proteins from other plant species. Phylogenetic analyses were performed using the neighbor-joining method by the MEGA version 5 program. The numbers next to the nodes are bootstrap values from 1,000 replicates. Black solid circles represent that these MYB proteins are related to flavonoid biosynthesis. GenBank accession numbers of R2R3-MYB proteins from other plant species are as follows: ZmLc, NP_001105339; PhAN1, AAG25928; VvMYC1, ACC68685; MdbHLH3, ADL36597; AtGL3: Q9FN69; VvMYCA1, ABM92332; MdbHLH33, ABB84474; PhJAF13, AAC39455; FaMYC1, AFL02462; FabHLH3, QIZ03068; FabHLH33, QIZ03069; AmDELILA: AAA32663; AtMYC1: BAA11933; AtEGL3: NP_176552; AtTT8: CAC14865; ZmB: CAA40544.


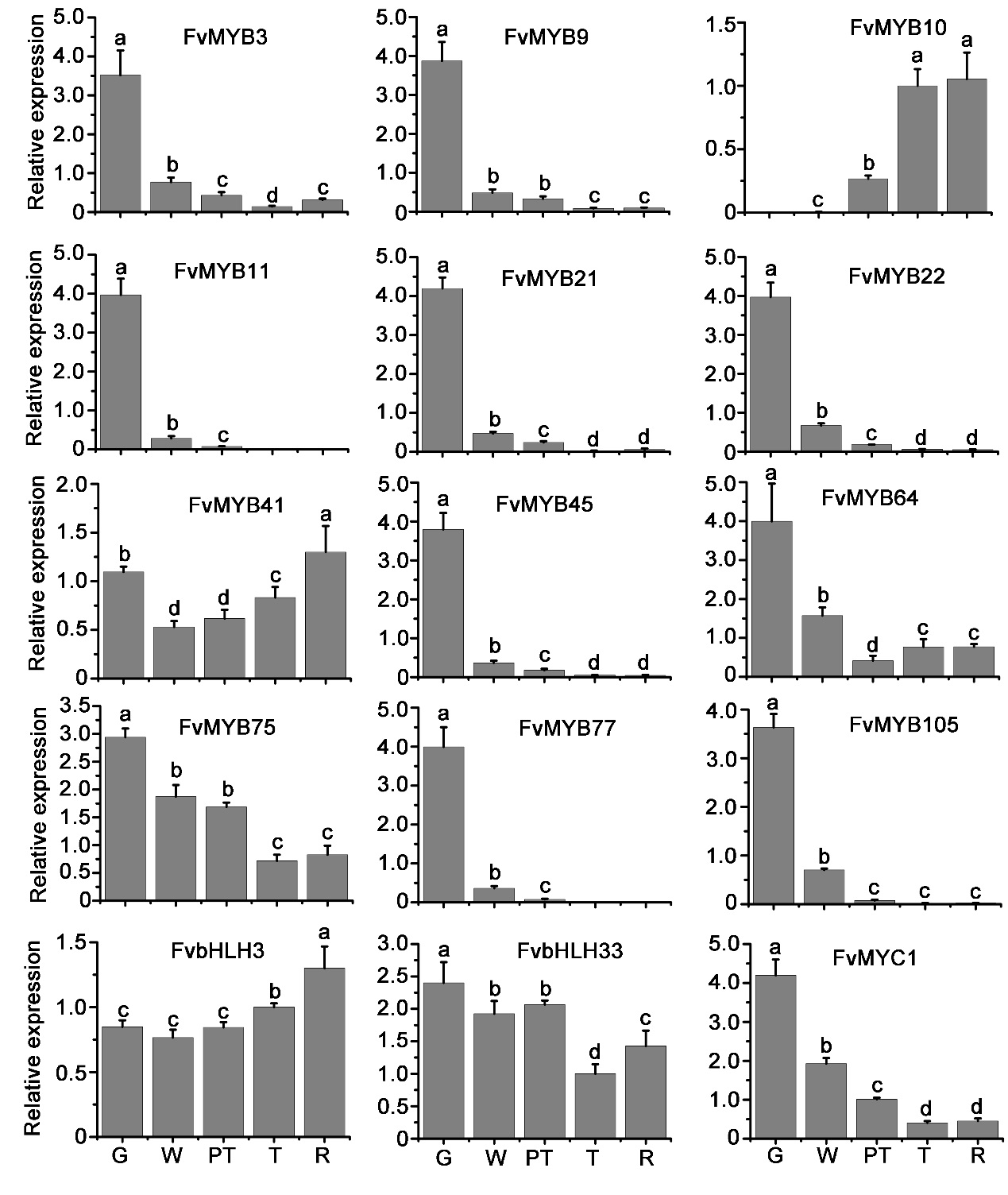


Supplementary Figure 3. Relative expression levels of *MYB* and *bHLH* genes in fruits of different development stages. Values were normalized to the expression level of internal control, and *FvActin* was used as the internal control. Data are presented as the mean ± SD (n = 3). G: green stage; W: white stage; PT: pre-turning stage; T: turning stage; R: ripe stage. The letters a to d indicate statistically significant differences, as determined by Tukey’s LSD test (P ≤0.05).


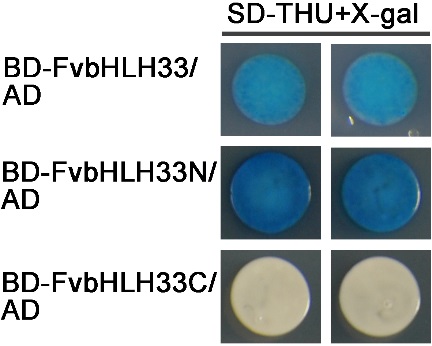


Supplementary Figure 4. FvbHLH33 had transcription activation activity in yeast cells. LexA yeast one-hybrid assay showing FvbHLH33 and N terminal of FvbHLH33 had transcription activation capacity while the C terminal of FvbHLH33 did not function as a transcription activator. Yeast cells co-expressing the indicated combinations of proteins were grown on selective medium (SD-Trp, -His, -Ura; SD-THU) with 80 mg/L X-gal. N terminal: 1-300 amino acids; C terminal: 301-643 amino acids.


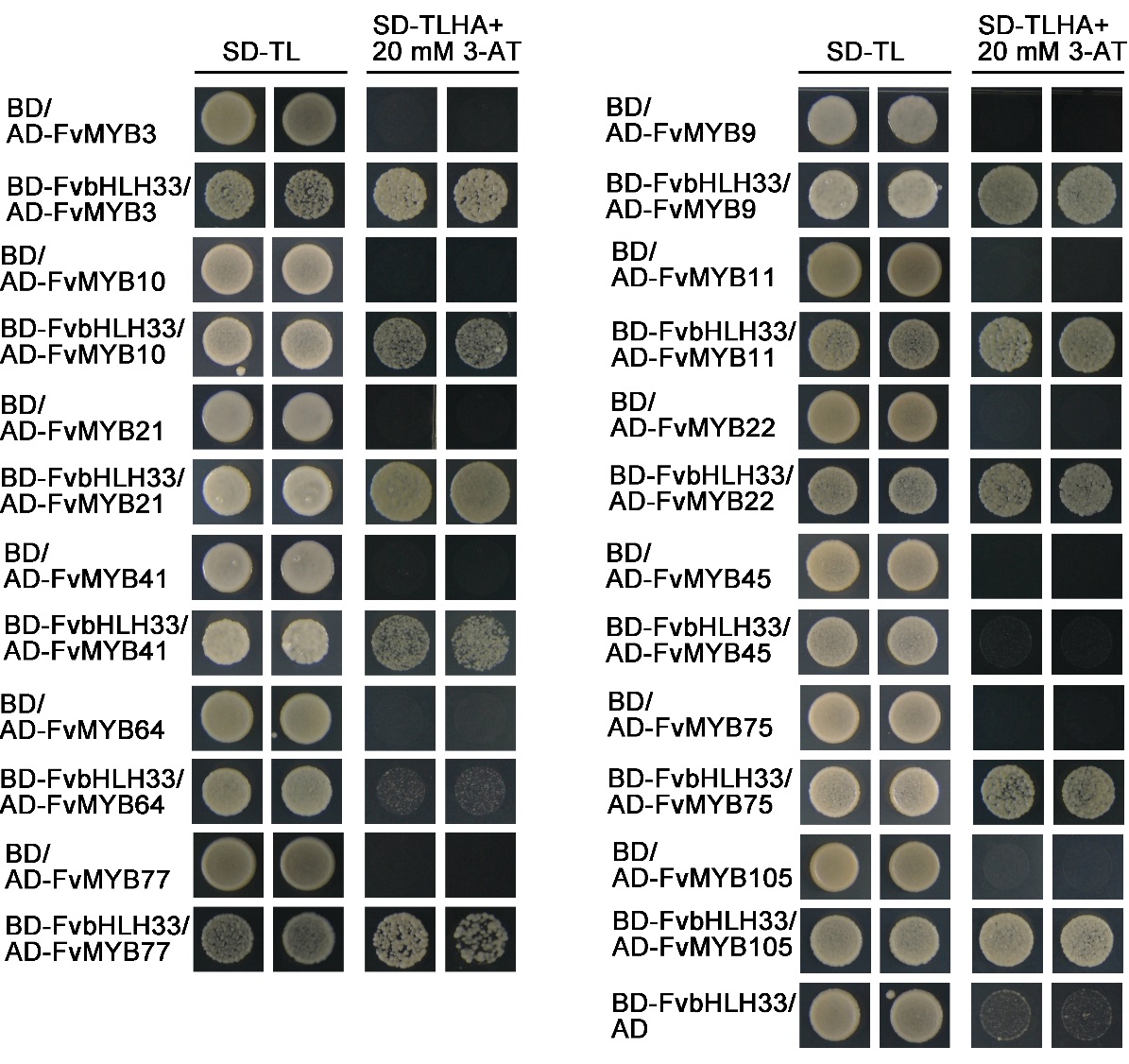


Supplementary Figure 5. The interaction of MYB and bHLH protein in yeast cells. GAL4 yeast two-hybrid assay showing interaction of 12 MYBs with FvbHLH33. Yeast cells co-expressing the indicated combinations of proteins were grown on non-selective (SD-Trp, -Leu; SD-TL) or selective media (SD-Trp, -Leu, -His, -Ade; SD-TLHA) with 20 mM 3-AT.


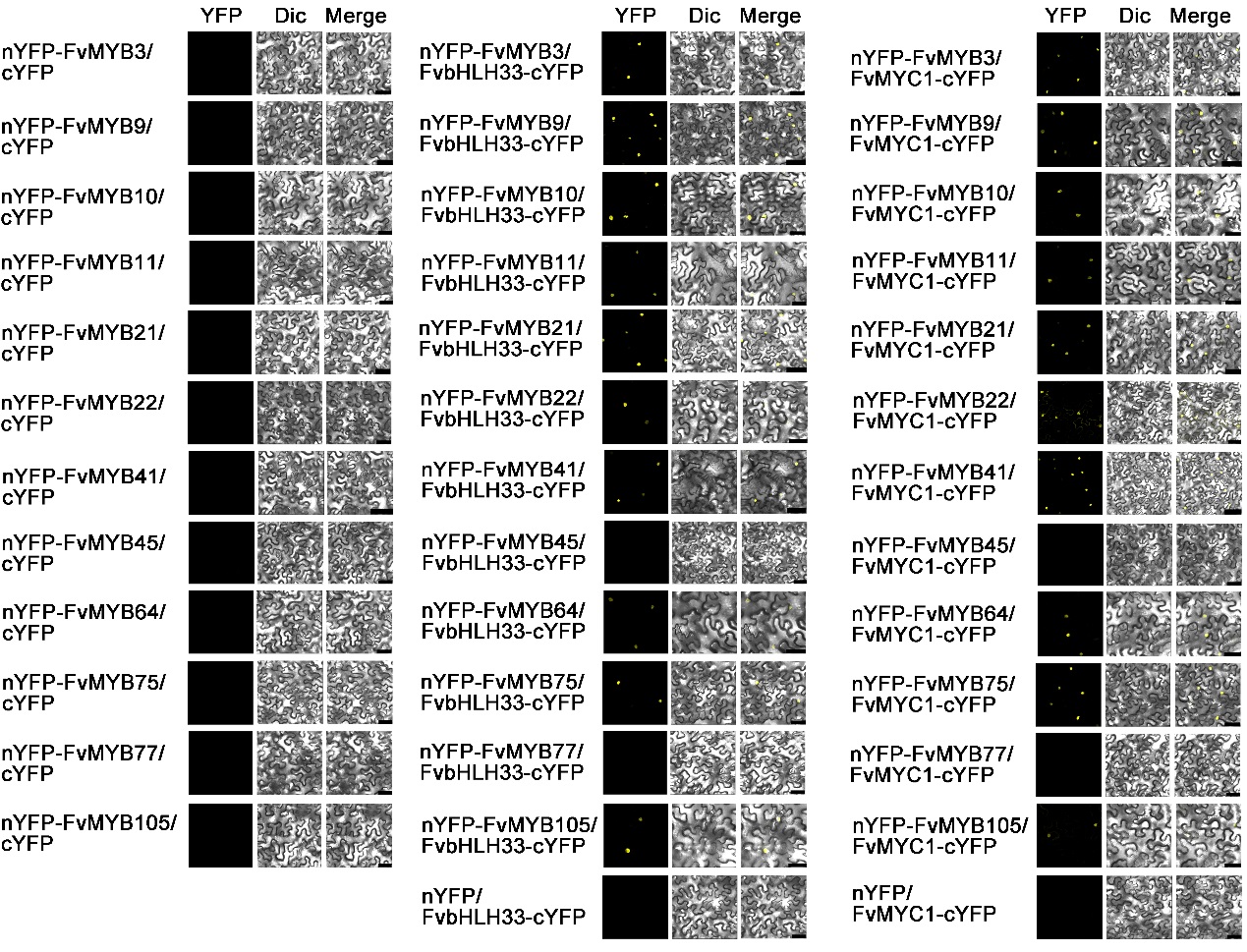


Supplementary Figure 6. BiFC assay showing interactions of FvbHLH33 and FvMYC1 with 12 MYB proteins in tobacco cells. The constructs encoding the indicated combinations of proteins were co-transformed into tobacco leaf epidermal cells. The images show overlays of fluorescence and light views. Dic, differential interference contrast. Bars, 50 μm.


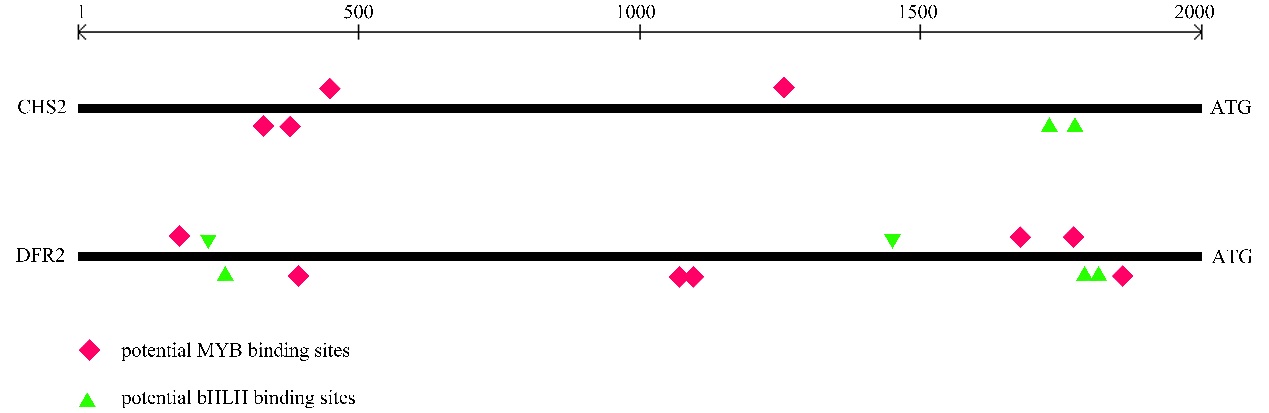


Supplementary Figure 7. Prediction of potential regulatory cis-elements in the promoter region of CHS2 and DFR2.


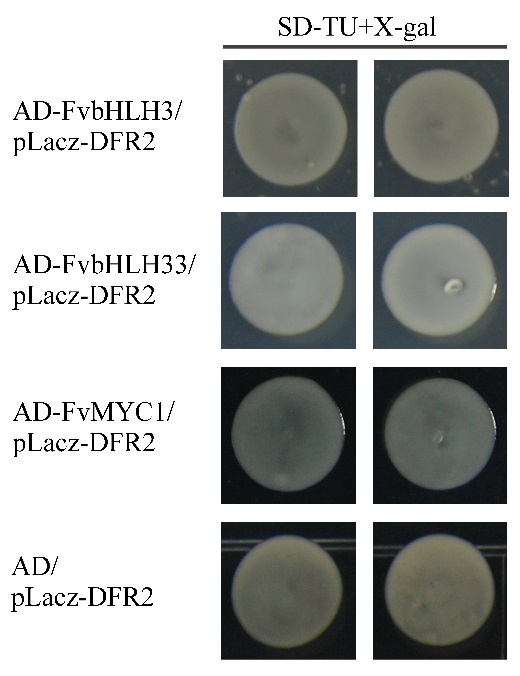


Supplementary Figure 8. Yeast one-hybrid showing no binding of bHLH proteins to DFR2 promoter. Yeast cells co-expressing the indicated combinations of proteins were grown on selective (SD-Trp, -Ura; SD-TU) with 80 mg/L X-gal.


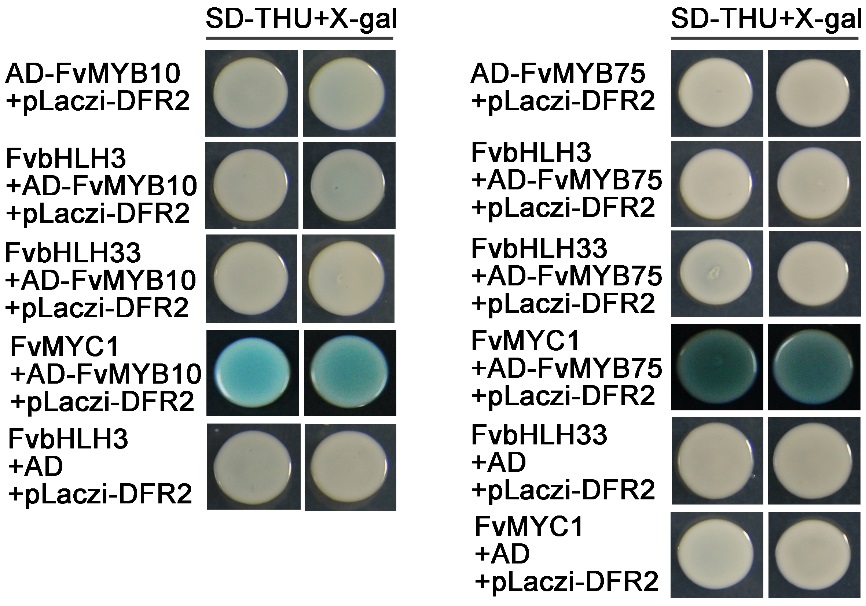


Supplementary Figure 9. The effect of interactions between MYB and bHLH proteins on the DFR2 promoter fused with *LacZ* reporter gene. Yeast cells co-expressing the indicated combinations of proteins were grown on selective medium (SD-Trp, -His, -Ura; SD-THU) with 80 mg/L X-gal. Blue precipitates represent cumulative β-galactosidase activity resulting from the activation of the *LacZ* reporter gene by binding.


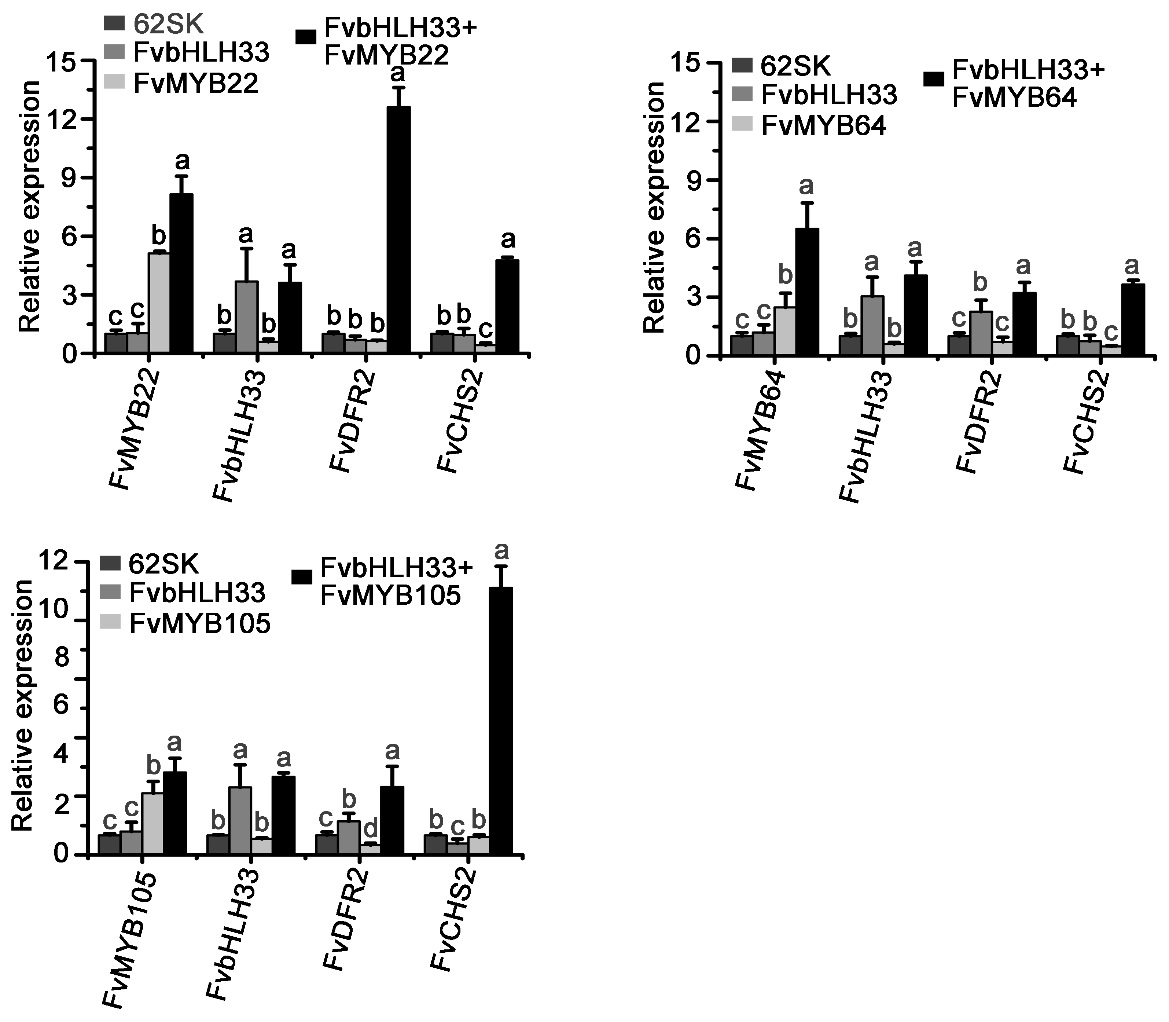


Supplementary Figure 10. Transcript levels of FvbHLH33, FvMYBs, FvCHS2 and FvDFR2 were analysed by qRT-PCR assays. *FvActin* was used as the internal control. Data are presented as the mean ± SD (n = 3). The letters a to c indicate statistically significant differences, as determined by Tukey’s LSD test (P ≤0.05).


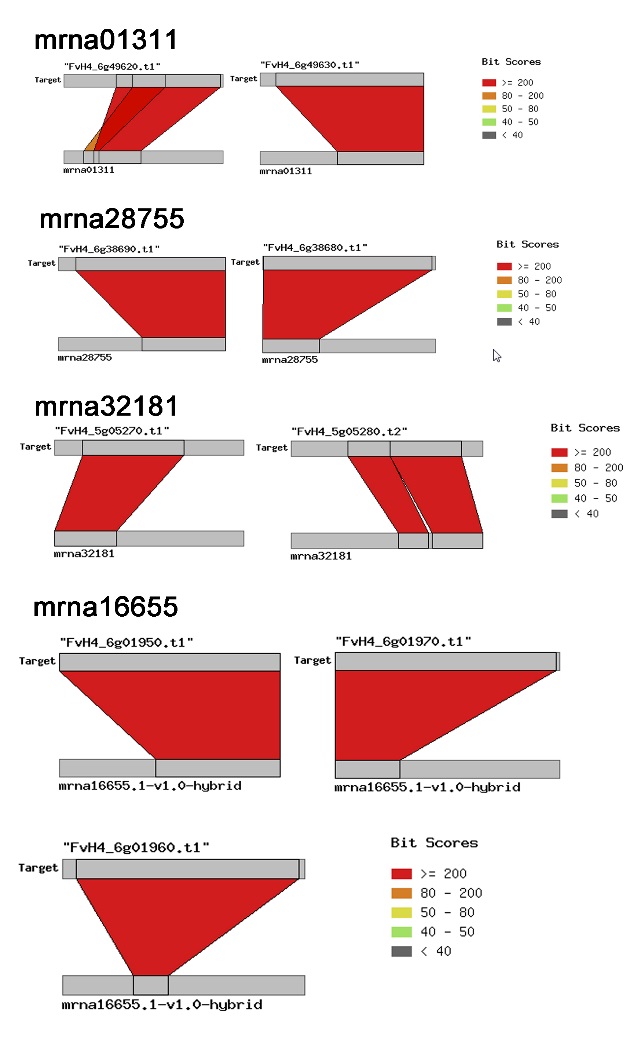


Supplementary Figure 11. The sequence blasting of R2R3-MYBs from previous study with strawberry genome version 4.0.a2. mrna32181, mrna16655, mrna01311 and mrna28755 were identified as R2R3-MYB from version 1.1.a2 by Li et al. When using these genes to blast against strawberry genome version 4.0.a2, we found that each of these four genes was annotated as two or three genes in version 4.0.2a, which suggested there were error annotations in version 1.1.2a.

Supplementary Figure 12. Protein sequence alignment of VvMYB5b, FnMYB5 and FvMYB41. Identical residues are shown in gray. The R2 domain and R3 domain represent the DNA binding domain corresponds to the R2R3-MYB repeats. The C1 and C3 motifs represent the conserved motifs in C-terminal regions.


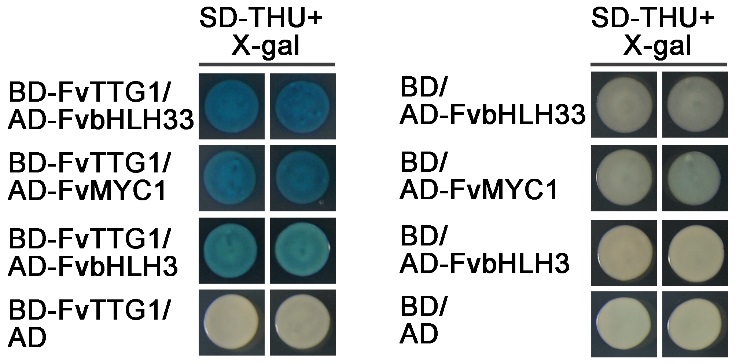


Supplementary Figure 13. The interaction of FvTTG1 and FvbHLH3, FvbHLH33 or FvMYC1 proteins in yeast cells. Yeast cells co-expressing the indicated combinations of proteins were grown on selective medium (SD-Trp, -His, -Ura; SD-THU) with 80 mg/L X-gal. Blue precipitates represent cumulative β-galactosidase activity resulting from the activation of the LacZ reporter gene by interacting proteins.

Supplementary Figure 14. The sequence alignment between FaMYC1 and FvMYC1. FaMYC1 from octoploid strawberry was used in previous study ([Schaart et al., 2012](#_ENREF_39)). The GenBank accession number of FaMYC1 was JQ989283. FvMYC1 was used in our study, and the gene ID was FvH4_5g02520.

Supplementary Figure 15. The sequence alignment between FabHLH33 and FvbHLH33. FabHLH33 from octoploid strawberry was used in previous study ([Schaart et al., 2012](#_ENREF_39)). The GenBank accession number of FabHLH33 was JQ989286. FvbHLH33 was used in our study, and the gene ID was FvH4_7g14230.


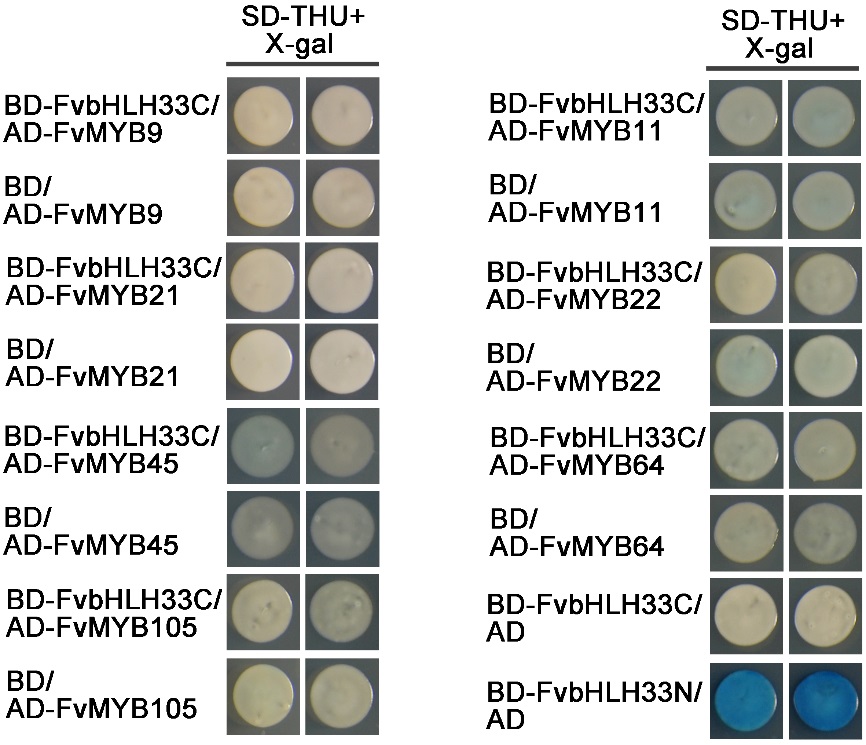


Supplementary Figure 16. The C terminal of FvbHLH33 cannot interact with MYB proteins. LexA yeast two-hybrid plate assays showing interactions of 7 FvMYBs and C terminal of FvbHLH33. Yeast cells co-expressing the indicated combinations of proteins were grown on selective medium (SD-Trp, -His, -Ura; SD-THU) with 80 mg/L X-gal. White precipitates indicate that no interaction has occurred. The self-activation of N terminal was served as positive control.


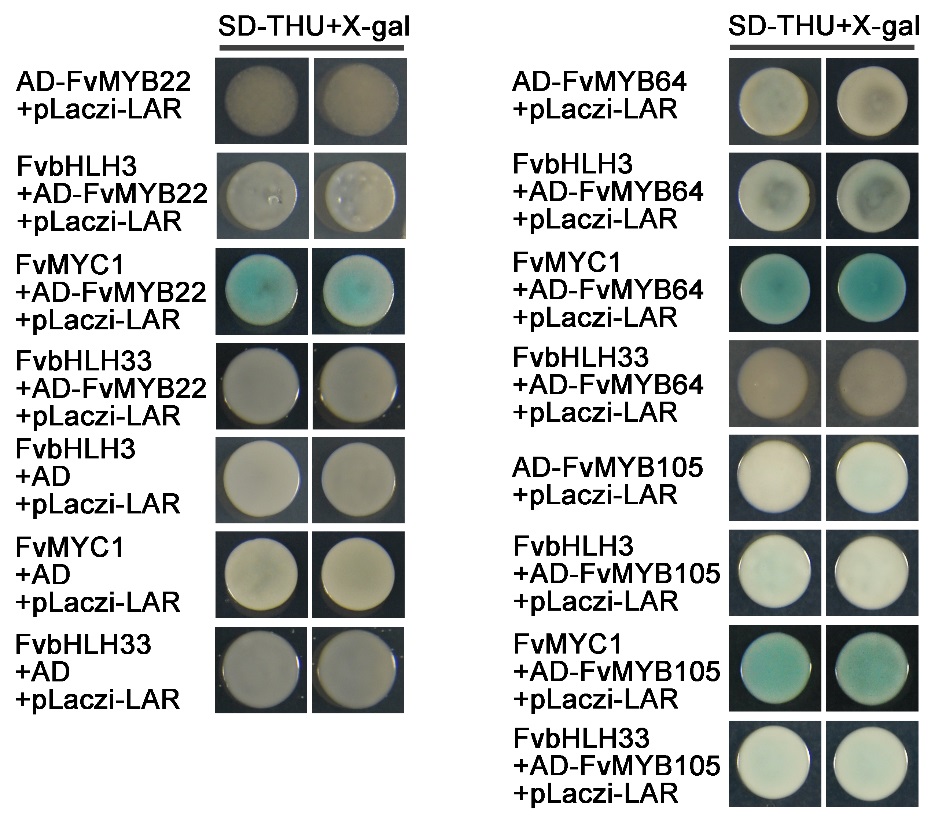


Supplementary Figure 17. The effect of interactions between MYB and bHLH proteins on the LAR promoter fused with *LacZ* reporter gene. Yeast cells co-expressing the indicated combinations of proteins were grown on selective medium (SD-Trp, -His, -Ura; SD-THU) with 80 mg/L X-gal. Blue precipitates represent cumulative β-galactosidase activity resulting from the activation of the *LacZ* reporter gene by binding.

Supplementary Table 1. The primer sequences used in vector construction.

Supplementary Table 2. The sequences of CHS2 and DFR2 promoters.

Supplementary Table 3. The primer sequences used in qRT-PCR assay.

Supplementary Table 4. The 118 MYB genes containing more than two MYB repeats in woodland strawberry.

Supplementary Table 5. The blast results of AtTT8, AtGL3 and AtEGL3 against the protein database of woodland strawberry.

Supplementary Table 6. The identity of protein sequences among FvMYB41, VvMYB5b and FnMYB5.
